# A Rare Case of Extensive Relapsing Disseminated Hydatid Disease with Multi-Organ Involvement: A Case Report

**DOI:** 10.3390/diseases14060215

**Published:** 2026-06-15

**Authors:** Silviu Gabriel Vlăsceanu, Radu Șerban Matache, Beatrice Mahler, Alexandru Stoichiță, Camelia Alexandra Paruschi, Alina Elena Tucana, Andrei Cristian Bobocea, Cornel Florentin Savu

**Affiliations:** 1 Department of Thoracic Surgery, “Marius Nasta” National Institute of Pneumology, 050150 Bucharest, Romania; silviu.vlasceanu@drd.umfcd.ro (S.G.V.); radu.matache@gmail.com (R.Ș.M.); camelia-alexandra.paruschi@rez.umfcd.ro (C.A.P.); alina-elena.tucana0226@rez.umfcd.ro (A.E.T.); andrei.bobocea@gmail.com (A.C.B.); drsavu25@yahoo.com (C.F.S.); 2 Thoracic Surgery I, University of Medicine and Pharmacy “Carol Davila”, 020021 Bucharest, Romania; 3 Department of Pneumology, “Marius Nasta” National Institute of Pneumology, 050150 Bucharest, Romania; 4 Pneumoftisiology II Discipline, Faculty of Medicine, University of Medicine and Pharmacy “Carol Davila”, 020021 Bucharest, Romania

**Keywords:** hydatid disease, echinococcosis, disseminated hydatid disease, mediastinum, pericardium, diaphragm, multidisciplinary team, *Echinococcus granulosus*, thoracic surgery, case report

## Abstract

Background: Hydatid disease, caused primarily by *Echinococcus granulosus*, remains a significant public health challenge in endemic regions. While hepatic (80–85%) and pulmonary (15–20%) involvements are common, multi-organ dissemination involving rare sites such as the pericardium, diaphragm, and mediastinum occurs in less than 0.1–2% of cases. Case presentation: We present a rare case of a 26-year-old male, a farmer for 10 years, with occupational exposure to dogs and horses, with a personal history of multiple surgically treated abdominal cysts in 2016, admitted after abdominal computed tomography revealed liver cysts greater than 5 cm, as well as mediastinal and diaphragmatic cysts. Histopathological examination of the surgically resected hepatic cyst material confirmed echinococcosis. Serology was also positive for echinococcosis. Echocardiography revealed a pericardial cyst, posterior to the left atrium. Under these circumstances, antiparasitic treatment was initiated by an infectious disease specialist, followed by surgical treatment of the abdominal cysts, confirming the final diagnosis of hydatid disease, and subsequently, surgical treatment of the thoracic hydatid cysts was performed. The postoperative course was complicated by bronchial superinfection with *Stenotrophomonas maltophilia*, identified from bronchial aspirate culture after extended incubation and managed with trimethoprim–sulfamethoxazole. Conclusions: This case underscores the necessity of lifelong surveillance in hydatid disease, the potential role of postoperative antiparasitic therapy in preventing long-term recurrence, and the vital role of a multidisciplinary team in managing complex, disseminated relapses.

## 1. Introduction

Echinococcal disease, or hydatidosis, is a parasitic disease caused by the tapeworm *Echinococcus*, divided into four species relevant to human infection: *E. granulosus* and *E. multilocularis*, which are the most common, causing cystic and alveolar echinococcosis, and two other species, *E. vogeli* and *E. oligarthrus*, causing polycystic echinococcosis, which are less frequent in human infection [1,2]. This disease is highly endemic in several regions worldwide and represents a serious public health concern in South America, the Middle East, the Eastern Mediterranean, sub-Saharan African countries, Russia, western China, Australia and New Zealand. The majority of cases reported in Central Europe and the USA occur in immigrants from endemic areas [3]. The disease is prevalent in areas where close contact with dogs occurs [4].

The evolution of the disease is typically slow, and the majority of cases remain asymptomatic for a prolonged period. Mortality (2–4%) is usually attributed to complications [1]. The clinical manifestations depend on the size, location, and the involved organ. Small cysts may remain asymptomatic for extended periods, while large cysts can compress adjacent organs, obstruct blood and lymphatic flow, and develop complications such as rupture and secondary infection. The growth rate of cysts is approximately 1–5 cm per year. The diagnosis of hydatid disease relies on serological methods, including serum antibody testing, and imaging modalities such as plain radiography, computed tomography (CT), and ultrasonography, combined with a detailed history of contact with infected dogs [5,6]. Current management strategies favor a combination of antiparasitic therapy and surgical resection to prevent rupture and recurrence [2,7].

Herein, we report an exceptionally rare case of disseminated relapsing hydatid disease with simultaneous pulmonary, pericardial, mediastinal, diaphragmatic, and hepatic involvement, managed successfully through a staged multidisciplinary surgical approach.

## 2. Case Presentation

We present the case of a 26-year-old male, a former smoker (7 pack-year smoking history, cessation 3 years prior) and former cannabis consumer (cessation 3 years prior), working as a farmer for 10 years, with occupational exposure to dust and fertilizers and contact with dogs and horses. His personal history was significant for multiple abdominal hydatid cysts (greater omentum, retrogastric space, liver segments II/III, spleen, right paracolic gutter, and Douglas space), previously treated surgically in 2016 (total omentectomy, splenectomy, liver segmentectomy with hepatic suture, and total cyst resection). Notably, in the interval between the initial surgery in 2016 and his current presentation, the patient received no antiparasitic therapy and no dedicated parasitological follow-up or imaging surveillance for echinococcosis, despite attending routine medical check-ups during this period. The exact serological assay, antibody titer, and laboratory reference range could not be retrieved from the available medical records and are therefore acknowledged as a limitation of the present report.

The patient presented with right upper quadrant pain and nausea, and independently decided to undergo abdominal computed tomography in February 2024. The investigation revealed multiple mediastinal cystic formations measuring 6.5 cm × 4 cm and 7.2 cm × 5.1 cm. Subdiaphragmatically, on the left side, cystic images were identified in the left hepatic lobe and the fundic region of the stomach, measuring 4.3 cm × 2.8 cm. On the right side, supradiaphragmatic cystic formations with internal septations were noted, measuring 6 cm × 4.5 cm and 5 cm × 5.7 cm. The liver also presented multiple cysts, with the largest measuring 14 cm × 12.3 cm in the right lobe Figure 1.

In light of these findings, the patient was referred to the infectious disease department, where antiparasitic therapy was initiated with Albendazole at a dose of 800 mg/day, administered as 400 mg twice daily, for 3 months, with monitoring of liver transaminases and a complete blood count every 2 weeks. Three weeks later, the patient was admitted to the General Surgery Department. Detailed baseline eosinophil count, liver function tests, and serial laboratory values during albendazole therapy could not be fully retrieved from the available records. However, no clinically significant hepatotoxicity or hematological toxicity requiring albendazole discontinuation was documented. Histopathological examination of surgically obtained hepatic cyst material confirmed the diagnosis of echinococcosis, which was further supported by positive serology. The patient subsequently underwent surgical management of one hepatic hydatid cyst. The procedure consisted of cyst inactivation, polycystectomy, evacuation of cyst contents, excision of the germinal layer, cavity drainage, and peritoneal drainage. Histopathological examination of the surgically obtained cyst material confirmed hydatid disease, showing the characteristic laminated membrane and pericystic host reaction (Figure 2). 

Following discharge from the general surgery department, the patient was admitted to our thoracic surgery department for further evaluation and management of the thoracic hydatid cysts. Thoracic computed tomography was performed (Figure 3), revealing multiple cystic formations, with the largest measuring 7 cm × 3.8 cm, located in the right paramediastinal region, retrocardiac space, and the right lower lobe of the lung.

From a respiratory standpoint, the patient was asymptomatic at presentation. Echocardiography revealed a cystic image measuring 5 cm, located posterior to the left atrium, well-defined, with no alterations in cardiac function Figure 3. Intraoperative findings confirmed that this lesion involved the pericardial compartment and was not merely a mediastinal cyst adjacent to the pericardium; therefore, it was classified as a pericardial hydatid cyst. On physical examination at admission to the thoracic surgery department, the patient was in good general condition, hemodynamically and respiratorily stable, and afebrile. Abdominal examination revealed a right subdiaphragmatic drain with an output of approximately 150 mL/24 h of bilious fluid, a soft abdomen without signs of peritoneal irritation, and a healing postoperative scar with normal appearance.

Preoperative workup included pulmonary function tests and diffusing capacity of the lungs for carbon monoxide (DLCO), both of which were within normal limits, and electrocardiography, which revealed sinus rhythm at a rate of 99 bpm. Blood tests demonstrated mild leukocytosis and an inflammatory syndrome at admission, which showed progressive improvement under empirical antibiotic therapy with cefazolin and metronidazole prior to surgical intervention.

On 17 April 2024, under general anesthesia with selective single-lung intubation, right-sided surgical treatment was performed via thoracotomy and included cystectomy of one diaphragmatic hydatid cyst and one pericardial hydatid cyst located posterior to the left atrium. Complete macroscopic resection was achieved. Histopathological evaluation of the thoracic resected specimens further corroborated the diagnosis, highlighting abundant host-derived fibrous tissue of the pericyst intimately adjacent to the diaphragmatic muscle bundles (Figure 4). Postoperative supportive treatment included anticoagulant therapy, corticosteroids, antibiotic therapy, anthelmintic therapy with albendazole 800 mg/day (400 mg twice daily, continued from the preoperative course), and analgesics.

On postoperative day 2, the patient was transferred to the thoracic surgery ward in fair general condition: afebrile and normotensive but tachycardic, without respiratory complaints. Thoracic drains showed no air leaks, with a cumulative serosanguineous output of 200 mL. The peritoneal drain yielded approximately 1000 mL of bilious fluid. Laboratory findings revealed worsening of the infectious syndrome: leukocytosis of 28,000/mm^3^, procalcitonin of 0.55 ng/mL, and C-reactive protein of 250 mg/L. In the context of the worsening infectious syndrome, microbiological investigations were initiated. Because of the postoperative inflammatory deterioration after complex thoracic surgery, bronchial superinfection was considered early in the differential diagnosis, and bronchial aspirate cultures were obtained to identify a potential respiratory pathogen.

Two flexible bronchoscopies were performed. The first, on 18 April 2024, revealed diffuse bilateral bronchitis with moderate mucous secretions and a small clot at the tracheal carina, which was aspirated; a BioFire respiratory panel returned negative. The second bronchoscopy, performed on 24 April 2024, confirmed diffuse bilateral bronchitis with mild extrinsic compression at a second-order subsegmental bronchus of the right lower lobe lateral basal segment, without mucopurulent secretions.

Given the worsening infectious syndrome, a repeat echocardiography was performed, which confirmed preserved global left ventricular systolic function, no significant valvulopathies, no pulmonary hypertension, and no lesions suggestive of endocarditis. Following infectious disease consultation, antibiotic therapy was adjusted: linezolid, meropenem, and fluconazole were replaced by vancomycin in combination with meropenem, with subsequent progressive improvement of the inflammatory syndrome and resolution of leukocytosis.

Notably, the bronchial aspirate cultures from the second bronchoscopy required an extended incubation period. The presence of Stenotrophomonas maltophilia and its sensitivity to Trimethoprim–Sulfamethoxazole (TMP-SMX) were only confirmed five days later, shortly before discharge. Consequently, targeted therapy with TMP-SMX was not initiated during hospitalization but was instead prescribed as part of the discharge regimen to ensure complete eradication of the pathogen.

The postoperative course was favorable; the posterior thoracic drain was removed on postoperative day 12, in the absence of air leaks or significant fluid output; and the patient was discharged on 30 April 2024. Discharge medication included trimethoprim–sulfamethoxazole (400/80 mg, 2 tablets every 12 h for 3 days, followed by 1 tablet every 12 h for 10 days), naproxen/esomeprazole, and metamizole. Albendazole was also continued after discharge at a dose of 800 mg/day, administered as 400 mg twice daily, until completion of the planned 3-month perioperative antiparasitic course, in accordance with published international recommendations for cystic echinococcosis. Complete blood count and liver function tests were periodically monitored during treatment, and no clinically significant hepatotoxicity or hematological toxicity requiring treatment discontinuation was documented.

Follow-up was performed at 6 months post-surgery, including clinical examination, pulmonary function tests, and thoracic computed tomography, which demonstrated no evidence of local recurrence or residual cystic disease. Chest radiographs performed at routine intervals showed no surgical complications. However, structured long-term parasitological surveillance including serial serology and abdominal imaging could not be fully documented, as the patient continued follow-up in his home region. This represents a limitation of the present report.

The chronological progression of the clinical course is illustrated in Figure 5.

## 3. Discussion

Hydatidosis is a parasitic disease caused most commonly in humans by the larval form of *Echinococcus granulosus* [2]. Echinococcosis is typically transmitted via the fecal-oral route, through direct contact with dogs, or by ingestion of parasitic eggs through contaminated water, food, or soil. The eggs of the parasite reach the liver via the portal vein, which acts as the first filter, retaining approximately 75% of the embryos, followed by the lungs as the second filter [1,4].

Although hydatidosis has a long history dating from ancient times, it remains highly relevant today, with significant social and economic impact, affecting large populations across different parts of the world [3]. Multi-organ involvement occurs in 20–40% of cases [8]. It is estimated that 10–19% of the parasites bypass the hepatic filter and spread to other organs [1,3]. Regarding the distribution of unusual locations, pericardial involvement accounts for 0.5–2% of cases, with 60% of cardiac cases involving the left ventricle and only 10% representing pericardial cysts. Diaphragmatic involvement is reported in 0.6–16% of cases, and mediastinal involvement in 0.5–2.6% of cases in endemic areas, further illustrating the particularity of the present case [9,10,11,12].

The pathophysiology of *Echinococcus granulosus* is defined by its protracted developmental pace; given that hydatidosis follows an insidious evolutionary trajectory, the parasitic lesions achieve a long-term equilibrium with the host’s tissues, remaining well tolerated for extended durations. As a result, the transition to a symptomatic state typically occurs after a significant chronological lag, often manifesting several years post-inoculation [2]. Regarding intrapulmonary distribution, pulmonary hydatid cysts most frequently involve the right lower lobe, a pattern also observed in our patient [5,13]. Atypical localizations such as the pericardium, diaphragm, and mediastinum are clinically important because they may remain asymptomatic for long periods but can later cause compression, infection, rupture, anaphylaxis, or even life-threatening cardiac or vascular complications [8,13,14,15,16].

Pulmonary cysts may present with cough, dyspnea, chest pain, hemoptysis, or secondary infection, and rupture into the bronchial tree or pleural cavity may lead to expectoration of clear fluid containing scolices or membrane fragments [17,18]. Diagnosis is based on epidemiological exposure, clinical history, imaging findings, and serological testing. Ultrasonography, CT, and MRI are useful for defining cyst location, morphology, complications, and multi-organ extension, while histopathological confirmation is usually obtained from surgically resected material when available [2,5,6].

Ultrasonographically, cysts can be classified into 5 stages:

- Stage I—hypoechoic lesion with thin walls;
- Stage II—septate cystic lesion;
- Stage III—cystic lesion with daughter cysts;
- Stage IV—pseudotumoral lesion;
- Stage V—totally or partially calcified lesion (inactive cyst) [13,19].

Chest radiography may show oval or round, well-defined opacities surrounded by normal lung parenchyma, while larger cysts may cause mediastinal displacement. CT is particularly useful for defining cyst location, wall characteristics, anatomical relationships, and complications such as rupture or secondary infection [14,16,20,21]. Differential diagnosis includes lung abscess, cavitary tuberculosis, benign cysts, neoplastic lesions, metastases, pulmonary hematoma, inflammatory masses, and hepatic lesions such as hemangioma or hepatocellular carcinoma [22,23]. Features supporting hydatid disease include epidemiological exposure, direct contact with dogs, positive *Echinococcus* serology, characteristic imaging findings, and, in ruptured pulmonary cysts, expectoration of clear fluid containing scolices or membrane fragments. Treatment usually combines antiparasitic therapy with surgical resection or, in selected cases, percutaneous aspiration and instillation of scolicidal agents [2,7,9]. Only a few comparable cases of disseminated multi-organ hydatidosis have been reported. Galvis et al. described a 32-year-old patient with thoracic and hepatic involvement requiring staged surgical management, including resection of lung, diaphragmatic, pericardial, mediastinal, and hepatic cysts [24]. Dantis and Gupta reported a 24-year-old patient with cystic lesions involving the mediastinum, lung, pericardium, diaphragm, chest wall, and liver [25]. Another study reports the case of a 56-year-old patient presenting to the emergency department with a 4-month history of dyspnea. Imaging investigations revealed a middle mediastinal mass, a cystic mass compressing the right pulmonary artery and the left atrium, and two multi-loculated hepatic cystic masses [26].

A comparative summary of the reported cases of disseminated hydatid disease with multi-organ involvement, including the present case, is presented in Table 1.

The present case is notable for several reasons that distinguish it from previously reported cases of disseminated hydatid disease. To our knowledge, this is the only reported case featuring a documented history of prior surgical treatment for abdominal hydatidosis, with subsequent relapse and multi-organ dissemination eight years later, suggesting that the 2024 presentation may represent either late recurrence due to residual viable parasitic elements after the 2016 surgery or a new infection/re-infestation related to the patient’s continued occupational exposure. Because no molecular comparison between the 2016 and 2024 lesions was available, the exact mechanism cannot be determined. Recurrence may have resulted from residual viable germinal tissue or perioperative spillage during the initial operation, particularly in the absence of documented postoperative antiparasitic therapy and structured echinococcosis surveillance. Conversely, reinfection remains plausible because the patient continued to work as a farmer and had ongoing exposure to dogs and livestock. For this reason, the present case should be interpreted as late relapsing or recurrent disseminated hydatid disease while acknowledging that reinfection cannot be excluded.

Furthermore, the present case is unique in its comprehensive diagnostic workup, combining thoraco-abdominal computed tomography, positive serology, histopathological examination of surgically obtained hepatic cyst material, and bronchoscopy, as well as in its documented microbiological complication and six-month follow-up without evidence of recurrence. These features, alongside the involvement of six distinct anatomical compartments, the lung, pericardium, mediastinum, diaphragm, liver, and multiple abdominal organs, underscore the exceptional complexity of this case.

The successful management of this case was contingent upon the coordinated involvement of multiple medical specialties. The infectious disease department initiated and monitored antiparasitic therapy; general surgery addressed the abdominal compartment; thoracic surgery managed the pulmonary, pericardial, mediastinal, and diaphragmatic cysts; cardiology provided essential preoperative risk assessment; pathology confirmed the histopathological diagnosis; and microbiology identified and guided the treatment of the postoperative superinfection. This case therefore exemplifies the indispensable role of a multidisciplinary team in the management of complex, disseminated hydatid disease.

The decision to proceed with a staged surgical approach addressing the abdominal compartment first, followed by thoracic surgery was guided by several considerations. Priority was given to the abdominal intervention, as the largest cyst (14 × 12.3 cm in the right hepatic lobe) posed the greatest risk of rupture and anaphylactic complication. Additionally, histopathological confirmation of the diagnosis via histopathological examination of surgically obtained hepatic cyst material was obtained during the abdominal intervention, prior to thoracic surgery. The interval between the two procedures allowed for clinical stabilization of the patient and optimization of antiparasitic therapy, ultimately contributing to the favorable surgical outcome. The thoracic stage was particularly challenging because the hydatid lesions involved anatomically sensitive compartments, including the mediastinum, diaphragm, lung parenchyma, and a cystic lesion in close relationship with the pericardium and left atrium. These locations increase the risk of intraoperative rupture, contamination of the operative field, bleeding, bronchopleural communication, cardiac compression, and incomplete resection. The surgical objective was therefore not only complete macroscopic removal, but also prevention of spillage and preservation of cardiopulmonary function.

The recurrence rate following surgical treatment is 4.65–22% [27]. This is particularly relevant in the present case, where multi-organ dissemination occurred eight years after the initial surgical treatment, likely reflecting incomplete eradication or perioperative spillage at the time of the first intervention. The absence of postoperative antiparasitic therapy and dedicated parasitological surveillance during the eight-year interval following the initial surgery may represent an additional contributing risk factor, potentially facilitating reactivation of residual germinal tissue or re-infestation given the patient’s ongoing occupational exposure. It should be noted that the reasons for which antiparasitic therapy was not prescribed following the 2016 intervention remain unclear, as no documentation was available in this regard; this observation should therefore be interpreted with caution. These findings highlight the potential benefit of systematic adjuvant antiparasitic therapy following surgical treatment of hydatid disease, as well as the role of structured long-term follow-up within a multidisciplinary framework.

Of particular interest in the present case is the postoperative identification of *Stenotrophomonas maltophilia* in the bronchial aspirate. *Stenotrophomonas maltophilia* is an opportunistic Gram-negative bacterium increasingly recognized as a nosocomial pathogen, particularly in immunocompromised patients and those with underlying pulmonary conditions. Its presence in this context may be attributed to the disruption of the bronchial epithelial barrier following thoracic surgery, combined with the immunosuppressive effect of chronic parasitic infection. In addition, other factors may have also contributed to Stenotrophomonas maltophilia colonization or infection, including exposure to the hospital environment during surgery and postoperative care, as well as prior broad-spectrum antimicrobial therapy, which may alter the respiratory microbiota and favor selection of opportunistic pathogens.

Trimethoprim–sulfamethoxazole (TMP-SMX) remains the first-line treatment of choice for *Stenotrophomonas maltophilia* infections, and its prompt initiation in this patient contributed to the favorable postoperative evolution.

This finding highlights the importance of routine microbiological surveillance in the postoperative management of thoracic hydatid disease. Also, preventive counseling should not be limited to clinical surveillance. In patients with ongoing occupational exposure, prevention of re-infestation should also include veterinary control and regular deworming of dogs, appropriate management of livestock, avoidance of feeding dogs with raw offal, hand hygiene after animal contact, and careful washing of potentially contaminated food.

This report has inherent limitations as a single case; causality between the absence of antiparasitic therapy and disease recurrence cannot be established, and whether the 2024 presentation represents reactivation or re-infestation remains speculative. In complex disseminated hydatid disease, follow-up should ideally include periodic clinical examination, thoracic and abdominal imaging, serological monitoring when available, and laboratory monitoring during antiparasitic therapy. Given the possibility of late recurrence, surveillance should extend beyond the early postoperative period, especially in patients with prior hydatid surgery, multi-organ disease, or persistent occupational exposure.

## 4. Conclusions

Hydatid disease may involve any organ, and disseminated multi-organ echinococcosis, although extremely rare, should be considered in patients with a history of prior hydatid surgery and occupational exposure. Early diagnosis and appropriate management are essential to prevent life-threatening complications that may arise during the course of the disease, such as compression, rupture, anaphylactic shock, and secondary infection. The case supports the need for clearly documented perioperative and postoperative antiparasitic strategies, including treatment duration and laboratory monitoring, given the potential role of albendazole in reducing recurrence risk. The present case further highlights the critical role of a multidisciplinary team encompassing thoracic surgery, general surgery, infectious disease, cardiology, pathology, and microbiology in ensuring coAmprehensive evaluation and optimal management of complex echinococcosis. Future efforts should focus on standardizing follow-up protocols and establishing international registries for disseminated hydatid disease, given its exceptional rarity and complex management requirements. Furthermore, public awareness regarding transmission, control, and prevention measures and the prophylactic anthelmintic treatment of sheep and dogs are of paramount importance in reducing the burden of this disease.

## Figures and Tables

**Figure 1:**
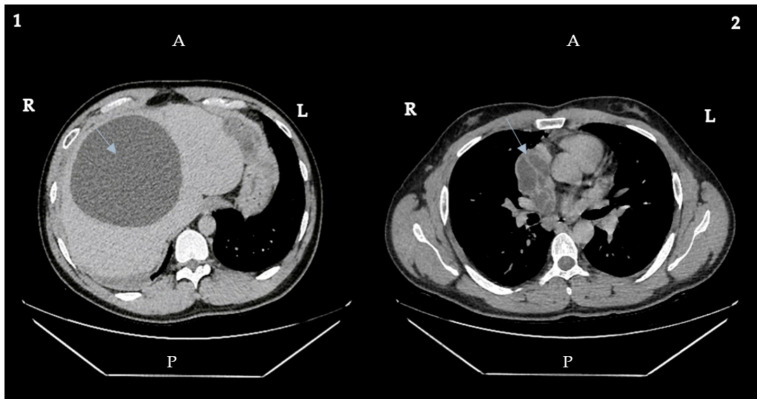
Thoraco-abdominal computed tomography (CT) findings: (**1**) axial CT image demonstrating a large, well-defined cystic lesion with a thin wall and no internal septations, located in the right hepatic lobe (arrow) and multiple other small hydatic cystic formations; (**2**) axial CT image showing oval, well-defined cystic formations with proligerous membranes exhibiting the “water lily sign,” consistent with ruptured hydatid cysts (arrowhead). Abbreviations: CT, computed tomography; R, right; L, left; A, anterior; P, posterior.

**Figure 2:**
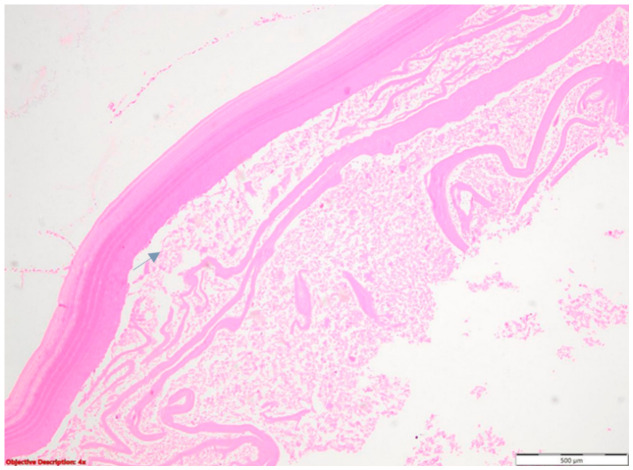
Histopathological appearance of the hydatid cyst wall: acellular eosinophilic laminated (outer) membrane (arrow), characteristic of Echinococcus granulosus; hematoxylin and eosin (HE) stain, original magnification ×40. Abbreviations: HE, hematoxylin and eosin.

**Figure 3:**
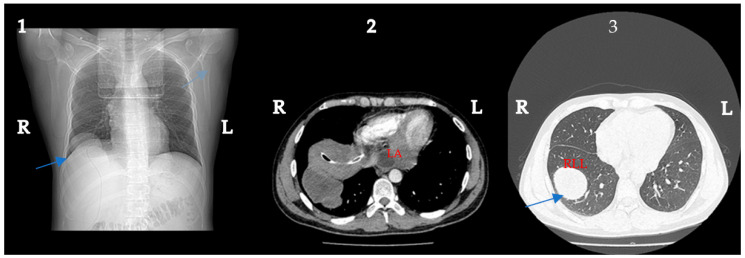
Thoracic computed tomography (CT) images obtained prior to thoracic surgery, following prior hepatic cystectomy (multiple hepatic hydatid cysts resected during the abdominal intervention): (**1**) axial CT image demonstrating a well-defined cystic lesion in the right lower lobe of the lung (arrow); note the abdominal hepatic drainage tube (biliary drain) visible in the inferior aspect of the image (arrowhead), confirming the staged surgical approach; (**2**) axial CT image showing a cystic lesion in the pericardium, posterior to the left atrium, consistent with a pericardial hydatid cyst (arrow); (3) axial CT image demonstrating an additional well-defined cystic lesion in the parenchyma of the right lower lobe (arrow). Abbreviations: CT, computed tomography; LA, left atrium; RLL, right lower lobe; R, right; L, left.

**Figure 4:**
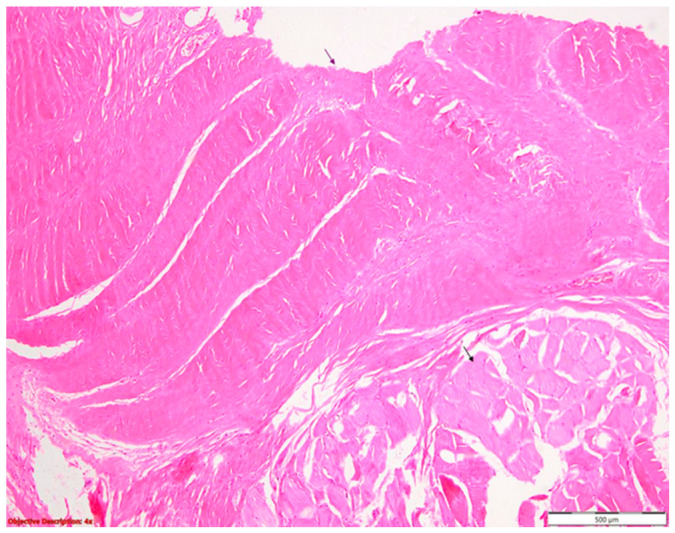
Histopathological appearance of the pericyst: abundant fibrous tissue of the host-derived pericyst (black arrow) adjacent to diaphragmatic muscle bundles (red arrow); hematoxylin and eosin (HE) stain, original magnification ×40. Abbreviations: HE, hematoxylin and eosin.

**Figure 5:**
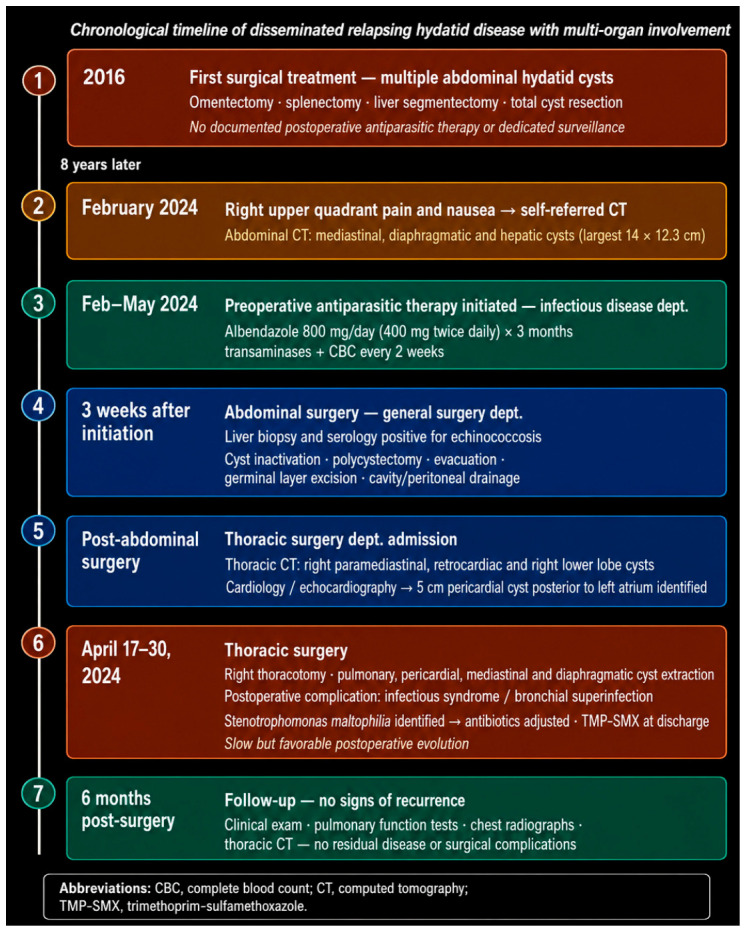
Chronological timeline of the clinical course of disseminated hydatid disease with multi-organ involvement.

**Table 1:** Comparative summary of reported cases of disseminated hydatid disease with multi-organ involvement.

Parameter	Galvis et al., 2023 [24]	Dantis & Gupta, 2022 [25]	Ramdani et al., 2021 [26]	Present Case, 2024
Age/sex	32 years/NR	24 years/NR	56 years/NR	26 years/male
Chief complaint	Abdominal pain (2 months)	Left-sided chest pain; left hemithorax heaviness	Dyspnea (4 months)	Right upper quadrant pain; nausea
Prior hydatid history	NR	NR	NR	Multiple abdominal cysts operated in 2016 (omentum, liver, spleen, Douglas space)
Organs involved	Lung, Diaphragm, Pericardium, Mediastinum, Liver	Mediastinum, Lung, Pericardium, Diaphragm, Chest wall, Liver	Mediastinum, Liver, Compression of right pulmonary artery and left atrium	Lung, Pericardium, Mediastinum, Diaphragm, Liver, Multiple abdominal
Diagnostic workup	CT imaging; serology	Contrast-enhanced CT thorax	CT imaging	Thoraco-abdominal CT; thoracic CT; serology (positive); surgically obtained hepatic cyst material; bronchoscopy
Serology	NR	NR	NR	Positive
Histopathology	Confirmed hydatid disease	NR	NR	Confirmed hydatid disease (surgical specimen—abdominal and thoracic)
Antiparasitic treatment	NR	NR	NR	Albendazole 800 mg/day × 3 months
Surgical approach	2 interventions: 1st—partial cyst resection (lung, diaphragm, pericardium, mediastinum) 2nd—major liver resection with extracorporeal circulation (retrohepatic vena cava infiltration)	Surgical resection Details not reported	Surgical resection Details not reported	2 interventions: 1st—abdominal (polycystectomy, splenectomy, omentectomy, liver segmentectomy) 2nd—thoracic (pulmonary, pericardial, mediastinal, diaphragmatic cyst extraction)
Postoperative complications	NR	NR	NR	*Stenotrophomonas maltophilia* bronchial superinfection → treated with TMP-SMX
Multidisciplinary team	NR	NR	NR	Yes: Thoracic surgery, general surgery, infectious disease, cardiology, pathology, microbiology
Outcome/ follow-up	Radical resection achieved; no follow-up reported	NR	NR	Complete surgical resection with no recurrence at 6-month follow-up.

NR = not reported; TMP-SMX = trimethoprim–sulfamethoxazole; CT = computed tomography. Reference numbers correspond to the numbering in the article’s reference list.

## Data Availability

No new data were created in this study. Data sharing is not applicable to this article as it is a case report; further data are available from the corresponding author upon reasonable request, subject to patient privacy restrictions.

## Abbreviations

The following abbreviations are used in this manuscript:

CT	Computed tomography
HE	Hematoxylin and eosin
TMP-SMX	Trimethoprim–sulfamethoxazole
NR	Not reported
MRI	Magnetic resonance imaging
WHO	World Health Organization
